# Supplementary material for: Effects of a Carbon Nanotube Additive on the Corrosion-Resistance and Heat-Dissipation Properties of Plasma Electrolytic Oxidation on AZ31 Magnesium Alloy
Source: Materials (Basel). 2018 Dec 2;11(12):2438. doi: 10.3390/ma11122438 (PMC6317174; doi:10.3390/ma11122438)
Supplement: Supplementary file 1 [file materials-11-02438-s001.pdf]

materials

Supplementary Figure

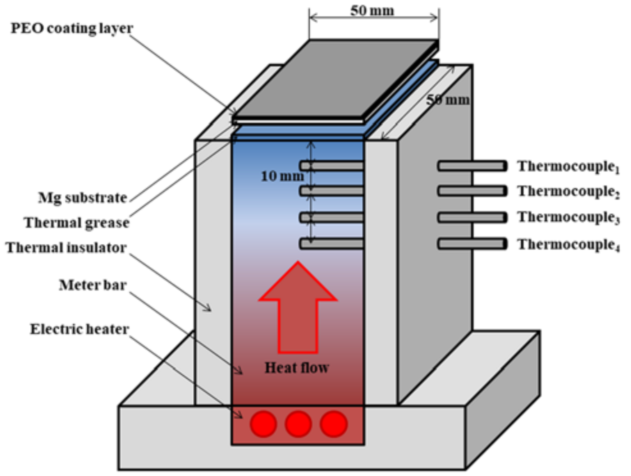

Figure S1. Schematic diagram of the heat flux measurement setup.

The heat flux measurement setup modified from the thermal conductivity measurement setup using a flow-meter method was used to measure the heat dissipation property in a previous study and shown in Figure S1 [1]. Stainless steel 304 block (thermal conductivity=16.2 $W \cdot m^{-1} \cdot k^{-1}$ at 200 ℃, POSCO, Korea) used in meter bar was cut to 50 x 50 x 120 mm, and was placed at the center of the setup. The heat loss of the meter bar was minimized by surrounding the heat insulator, and the heat source was inserted at the bottom center of the meter bar. The magnesium specimen was placed on the top of the meter bar with thermal grease (3.8 $W \cdot m^{-1} \cdot k^{-1}$, Evercool TC-200, Taiwan), which reduces the interfacial thermal resistance. Four T-type thermocouples are placed 10 mm apart from the top of the meter bar. Temperature changes were recorded on a monitoring system (MV 1000, Yokogawa, Japan). The temperature of the heat source was maintained at 200 ℃ during the measurement. After more than 30 minutes, the heat flux was calculated when the steady-state temperature was reached.

The heat from the heat source is transferred to the specimen through conduction and released to the atmosphere through the radiation of the specimen. Therefore, the dissipated heat flux can be calculated from the heat flux of the meter bar. The heat flux was calculated by the temperature of the four thermocouples and the thermal conductivity of the meter bar. The heat flux (Q_{xy}, W/m^2) between two thermo-couples (x and y) can be expressed as following equation [2,3]:

$$Q_{xy} = (\lambda \times A / d_{xy}) \times (T_x - T_y), \tag{1}$$

where λ, A, d_{xy}, T_x and T_y are the thermal conductivity of the meter bar (16.2 $W \cdot m^{-1} \cdot k^{-1}$), the cross-sectional area of the meter bar (25 cm^2), the distance between two thermocouples (10 mm), the temperatures of the any thermocouples x and y, repectively. That is, the heat flux can be obtained by the average of Q_{21} Q_{32} and Q_{43} through temperatures of four thermocouples (Thermocouple$_1$, Thermocouple$_2$, Thermocouple$_3$ and Thermocouple$_4$).

References

1. Lee, J.; Kim, D.; Choi, C.-H.; Chung, W. Nanoporous anodic alumina oxide layer and its sealing for the enhancement of radiative heat dissipation of aluminum alloy. *Nano Energy* **2017**, *31*, 504-513, doi:10.1016/j.nanoen.2016.12.007.
2. ASTM, D5470-06: Standard Test Method for Thermal Transmission Properties of Thermally Conductive Electrical Insulation Materials. *ASTM Int.* **2006**, doi:https://doi.org/10.1520/D5470-06.

3. Tan, J.C.; Tsipas, S.A.; Golosnoy, I.O.; Curran, J.A.; Paul, S.; Clyne, T.W. A steady-state Bi-substrate technique for measurement of the thermal conductivity of ceramic coatings. *Surface and Coatings Technology* **2006**, *201*, 1414-1420, doi:https://doi.org/10.1016/j.surfcoat.2006.02.010.
